# Synthesis and Biological Evaluation of Novel Furozan-Based Nitric Oxide-Releasing Derivatives of Oridonin as Potential Anti-Tumor Agents

**DOI:** 10.3390/molecules17067556

**Published:** 2012-06-18

**Authors:** Dahong Li, Lei Wang, Hao Cai, Yihua Zhang, Jinyi Xu

**Affiliations:** 1 Department of Medicinal Chemistry, China Pharmaceutical University, 24 Tongjia Xiang, Nanjing 210009, China; 2 State Key Laboratory of Natural Medicines, China Pharmaceutical University, 24 Tongjia Xiang, Nanjing 210009, China; 3 Center of Drug Discovery, China Pharmaceutical University, 24 Tongjia Xiang, Nanjing 210009, China

**Keywords:** NO donor, oridonin, hybrid, anti-tumoragents, SAR

## Abstract

To search for novel nitric oxide (NO) releasing anti-tumor agents, a series of novel furoxan/oridonin hybrids were designed and synthesized. Firstly, the nitrate/nitrite levels in the cell lysates were tested by a Griess assay and the results showed that these furoxan-based NO-releasing derivatives could produce high levels of NO *in vitro*. Then the anti-proliferative activity of these hybrids against four human cancer cell lines was also determined, among which, **9h** exhibited the most potential anti-tumor activity with IC_50_ values of 1.82 µM against K562, 1.81 µM against MGC-803 and 0.86 µM against Bel-7402, respectively. Preliminary structure-activity relationship was concluded based on the experimental data obtained. These results suggested that NO-donor/natural product hybrids may provide a promising approach for the discovery of novel anti-tumor agents.

## 1. Introduction

Nitric oxide, a special gaseous molecular, is a key mediator involved in many physiological and pathological processes [1,2]. High concentrations of NO and its metabolic derivatives can modify functional proteins, leading to cell cycle arrest and apoptosis, particularly in tumor cells [3,4,5]. Indeed, some synthesized NO-releasing compounds have shown cytotoxic activity against human carcinoma cells *in vitro* and inhibit the growth and metastasis of cancers *in vivo* [6,7,8]. So, the NO-based anti-cancer agents have been investigating for cancer therapy at clinic [9,10]. Furoxans represent one class of NO donors that can produce high levels of NO and exhibit strong anti-cancer activity [11,12]. In the previous work by our group, several classes of NO-releasing compounds have been reported, which possess strong anti-proliferative activity against human carcinoma cells *in vitro*, inhibition of cancer cells growth *in vivo* and the ability to increase sensitivity of Pgp-mediated multidrug resistance (MDR) in solid tumors, separately [13,14,15,16,17]. These results motivated us to further design some novel NO-donor/natural product hybrids.

**Figure 1:**
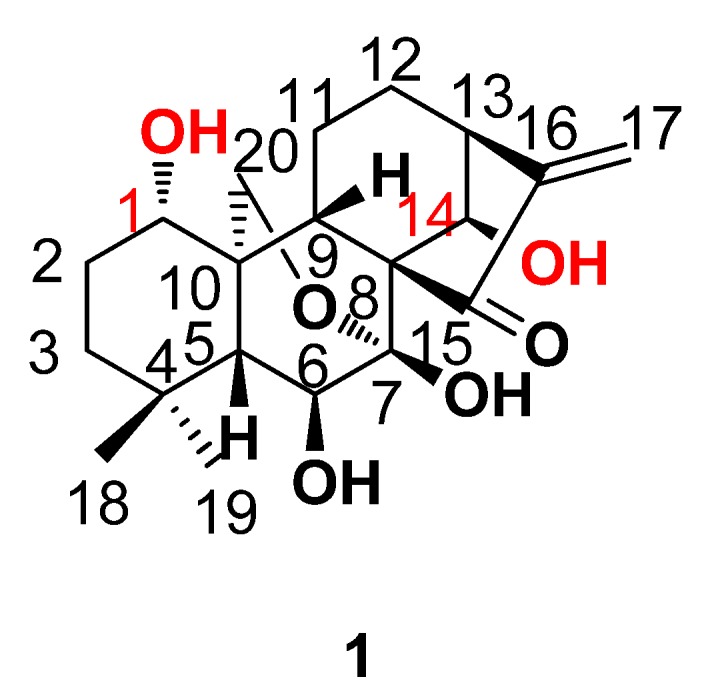
The structure and atom numbering of oridonin.

Oridonin (**1**, see Figure 1) is a commercially available natural *ent*-kaurene diterpenoid that has recently attracted much attention because of its anti-tumor activity with a mechanism of inhibition effect on nuclear factor κB (NF-κB) activation, induction of G_2_/M phase arrest and apoptosis [18]. Oridonin has been safely used for the treatment of hepatoma and promyelocytic leukemia in China for many years. In previous studies, we found that a series of novel 1-*O*- and 14-*O*-derivatives of oridonin exhibited stronger cytotoxicity against six cancer cell lines *in vitro* and some of them had stronger anti-tumor activity than the parent compound **1** and the positive control cyclophosphamide in mice with H22 liver tumor *in vivo* [19,20,21]. Hence, it may be a desired lead compound using for further design of novel furoxan-based NO-releasing derivatives for the development of anti-tumor agents. Therefore, a series of novel furozan-based nitric oxide-releasing derivatives of oridonin were designed and synthesized.

## 2. Results and Discussion

### 2.1. Synthesis of Furoxan-Based NO Donor

**Scheme 1:**
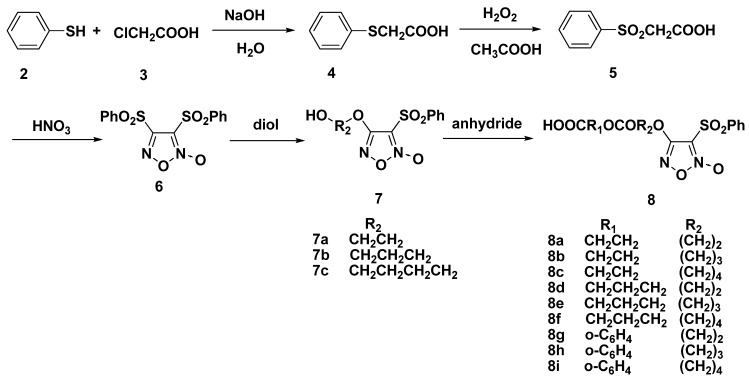
Synthesis of compounds **8a**–**i**.

The substituted furoxans were prepared in five steps in the following way (Scheme 1). The starting material benzenethiol (**2**) was converted to 2-(phenylthio)acetic acid (**4**) by treatment with chloroacetic acid (**3**). Then, compound **4** was oxidized by 30% H_2_O_2_ aqueous solution to give 2-(phenylsulfonyl) acetic acid (**5**) and fuming HNO_3_ was added to obtain diphenylsulfonylfuroxan (**6**), which was then converted to various monophenylsulfonylfuroxans **7a**–**c** by treatment with the corresponding diol. Finally, anhydrides were added and furoxan-based NO donors **8a**–**i** were obtained.

### 2.2. Synthesis of Furoxan/Oridonin Hybrids

**Scheme 2:**
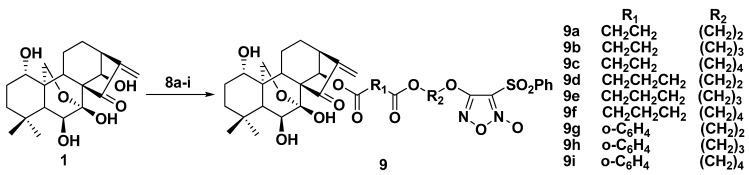
Synthesis of compounds **9a**–**i**.

The resulting furoxans **8a**–**i** were treated with oridonin to give the target compounds **9a**–**i**, as shown in Scheme 2.

**Scheme 3:**
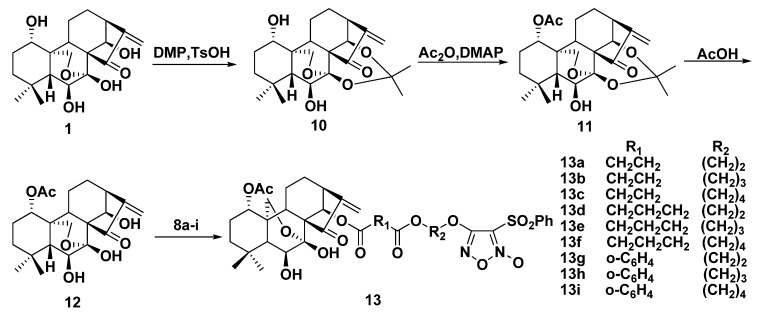
Synthesis of compounds **13a**–**i**.

Treatment of oridonin with 2,2-dimethoxypropane (DMP) in the presence of *p*-toluenesulfonic acid (TsOH) in acetone afforded 7,14-(1-methylethylene)-dioxyoridonin derivative **10**. Compound **10** upon reaction with Ac_2_O/pyridine yielded 1-*O*-acetyl derivative **11**. Deprotection of **11** with AcOH gave 1-*O*-acetyl-oridonin **12** in quantitative yield. Target compounds **13a**–**i** were prepared by reaction of **12** with furoxan-based NO donors **8a**–**i** in the presence of 4-dimethylaminopyridine/1-ethyl-3-(3-dimethyllaminopropyl)carbodiimide hydrochloride (DMAP/EDCI) in CH_2_Cl_2_, as shown in Scheme 3.

### 2.3. NO-Releasing Test of Hybrids ***9a**–**i*** and ***13a**–**i** in Vitro*

**Figure 2:**
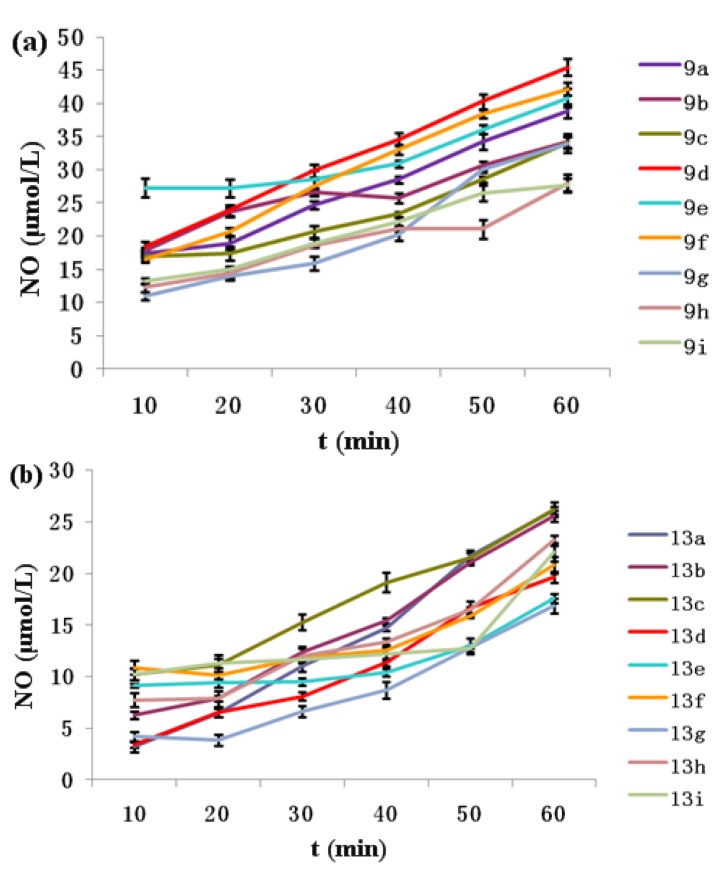
Variable levels of NO produced by compounds (**a**) **9a**–**i** and (**b**) **13a**–**i**.

The levels of nitrate/nitrite in the lysates of target compounds **9a**–**i** and **13a**–**i** were determined at 100 μM by Griess assay over a duration of 0–60 min. As shown in Figure 2, variable levels of NO were produced by compounds **9a**–**i** and **13a**–**i**. The concentration of NO increased with time, and at the time point of 60 min, all tested compounds produced more than 15 μmol/L of NO. The amount of NO released by compounds **13a**–**i** (**13g** with the lowest level of 16.88 μmol/L at the 60 min time point) was less than that of **9a**–**i** (**9d** with the highest level of 45.44 μmol/L at the 60 min time point). 

### 2.4. Anti-proliferative Activity *in Vitro*

The anti-proliferative activity of oridonin and its NO-donor hybrids **9a-**i as well as 1-*O*-derivatives of oridonin (**12**) and its NO-donor hybrids **13a**–**i** was evaluated against four human cancer cell lines (Bel-7402, K562, MGC-803, CaEs-17) by MTT assay. The results are shown in Table 1 [22]. All the target compounds **9a**–**i** and **13a**–**i** exhibited stronger anti-proliferative activity than their parent compounds **1** (oridonin) and **12** (1-oxo-oridonin), correspondingly. Among them, **13a**–**i** released less NO (Figure 2) and showed less potent anti-proliferative activity than **9a**–**i**. For example, **13e** with IC_50_ value of 2.13 µM compared to **9e** (1.33 µM) against Bel-7402 cells, **13g** with IC_50_ value of 2.45 µM compared to **9g** (1.33 µM) against MGC803 cells, and so on. These results and our previous studies [13,14,15,16,17] indicated that the releasing of NO contributed to anti-proliferative activity and higher levels of NO releasing could produce stronger activity. 

**Table 1:** IC_50_ values of the furoxan/oridonin hybrids against four human tumor cell lines *^a^*.

Compd.	Bel-7402	K562	MGC-803	CaEs-17
Taxol *^b^*	1.89 ± 0.09	0.41 ± 0.02 *^c^*	0.85 ± 0.06 *^c^*	0.43 ± 0.03 *^d^*
Oridonin	7.48 ± 0.53	4.76 ± 0.32	5.69 ± 0.39	11.03 ± 1.02
**9a**	2.37 ± 0.85	4.33 ± 0.14	3.22 ± 0.19	8.46 ± 0.05
**9b**	1.91 ± 0.09	3.46 ± 0.60	2.57 ± 0.07	6.98 ± 0.20
**9c**	2.23 ± 0.04	4.02 ± 0.05	3.46 ± 0.23	8.17 ± 1.01
**9d**	1.89 ± 0.22	3.78 ± 0.19	3.08 ± 0.47	8.04 ± 0.18
**9e**	1.33 ± 0.15	2.85 ± 0.03	2.21 ± 0.16	6.77 ± 0.32
**9f**	1.97 ± 0.04	3.72 ± 0.26	3.23 ± 0.25	8.09 ± 0.47
**9g**	0.95 ± 0.21 *^c^*	1.94 ± 0.14	1.98 ± 0.13	4.81 ± 0.10 *^c^*
**9h**	0.86 ± 0.08 *^c^*	1.82 ± 0.07	1.81 ± 0.20	4.56 ± 0.32 *^c^*
**9i**	0.97 ± 0.10 *^c^*	1.92 ± 0.34	1.90 ± 0.11	5.24 ± 0.18
**12**	3.21 ± 0.25	5.06 ± 0.18	4.05 ± 0.04	7.24 ± 0.41
**13a**	2.85 ± 0.14	4.65 ± 0.07	3.77 ± 0.31	5.30 ± 0.28
**13b**	2.19 ± 0.19	3.85 ± 0.06	2.90 ± 0.12	4.11 ± 0.07 *^c^*
**13c**	2.76 ± 0.42	4.11 ± 0.15	3.65 ± 0.40	5.22 ± 0.12
**13d**	2.70 ± 0.09	4.08 ± 0.30	3.64 ± 0.12	5.38 ± 0.24
**13e**	2.13 ± 0.17	3.04 ± 0.21	2.79 ± 0.10	4.00 ± 0.31 *^c^*
**13f**	2.66 ± 0.30	3.97 ± 0.16	3.42 ± 0.27	5.11 ± 0.39
**13g**	1.94 ± 0.13	2.22 ± 0.29	2.45 ± 0.51	3.28 ± 0.06 *^c^*
**13h**	1.72 ± 0.08	2.08 ± 0.34	2.22 ± 0.29	3.24 ± 0.23 *^c^*
**13i**	1.86 ± 0.15	2.65 ± 0.08	2.41 ± 0.16	3.13 ± 0.21 *^c^*

^a^ Results are expressed as IC_50_ values in µM and the values are means ± SD; n = 3. ^b^ Taxol was used as a positive control. *^c^* *p* < 0.05 *versus* oridonin; *^d^* *p* < 0.01 *versus* oridonin.

Among the tested compounds, the series **9g**–**i** and **13g**–**i** with a *o*-C_6_H_4_ linker (R_1_) (compounds **g**–**i**) showed lower IC_50_ values than the corresponding compounds **a**–**f**. Compared the IC_50_ values of the compounds of series **a**–**c** with **d**–**f** in different cell lines, there was a decline with the extension of the length of R_1_. In general, when R_1_ were aromatic groups (compounds **g**–**i**), the activity was stronger than those with alkyl groups. While R_1_ were alkyl groups, IC_50_ values decreased with lengthening of carbon chain. In almost all cases (except **13h**), when the length of R_2_ is three carbons, more potential anti-proliferative activity was observed than those of two and four carbons, correspondingly (for instance, **9b** > **9a** and **9c**, **9e** > **9d** and **9f**, **9h** > **9g** and **9i**, **13b** > **13a** and **13c**, **13e** > **13d** and **13f**). This suggested that the length of the linker group R_2_ with three carbons would be more suitable. In all the target synthetic hybrids, compound **9h** (R_1_=*o*-C_6_H_4_; R_2_=CH_2_CH_2_CH_2_) exhibited the most potential anti-tumor activity against tested cell lines: IC_50_ values of 0.86 µM against Bel-7402 (stronger than parent compound oridonin of 7.48 µM and positive control taxol of 1.89 µM), 1.82 µM against K562, 1.81 µM against MGC-803 (stronger than oridonin of 5.69 µM) and 4.56 µM against CaEs-17 (stronger than oridonin of 11.03 µM). Subsequent design and synthesis of novel NO releasing anti-tumor agents based on present SAR and more intensive biological studies were undertaking.

## 3. Experimental

### 3.1. Chemistry

All commercially available solvents and reagents were used without further purification. Melting points were taken on XT-4 micro melting point apparatus and are uncorrected. Infrared (IR) spectra (KBr pellets) were recorded on a Nicolet Impact 410 instrument (Madison, WI, USA). ^1^H-NMR spectra were recorded at 300 MHz with a Bruker AV-300 spectrometer (Karlsruhe, Germany) in the indicated solvents (TMS as internal standard): The values of the chemical shifts are expressed in *δ* values (ppm) and the coupling constants (*J*) in Hz. Mass spectra were obtained using FTMS-2000 (Madison, WI, USA). HR-MS were obtained using an Agilent QTOF 6520 (Palo Alto, CA, USA). Compounds **2**–**4** were commercially available. Compounds **5**, **6**, **7a**–**c**, **10**, **11** and **12** were synthesized, as previously described [13,19,20].

#### 3.1.1. General Procedure for the Preparation of ***8a**–**i***

Compound **7a**–**c** (2 mmol) in pyridine (5 mL) was mixed with the corresponding anhydride (4 mmol) by stirring at room temperature for 6–12 h. The mixture was concentrated *in vacuo*, dissolved in H_2_O (15 mL) and extracted with CH_2_Cl_2_ (15 mL × 3). The organic layers were combined, washed with water and saturated NaCl solution sequentially, dried over anhydrous Na_2_SO_4_, and concentrated *in vacuo*. The crude products **8a**–**i** used for the next step without further purification. 

#### 3.1.2. General Procedure for the Preparation of ***9a**–**i*** and ***13a**–**i***

Compounds **8a**–**i** (0.24 mmol) were dissolved in CH_2_Cl_2_ (10 mL) and stirred at room temperature. Oridonin or its derivative **12** (0.2 mmol), EDCI (93 mg, 0.6 mmol) and DMAP (catalytic amount) were added. After 8–12 h, the reaction mixture was washed with water and saturated NaCl solution sequentially, dried over anhydrous Na_2_SO_4_, and concentrated *in vacuo*. The crude products were purified by column chromatography (MeOH/CH_2_Cl_2_ 1:200 v/v) to give the title compounds.

*ent-1α,6β,7β-Trihydroxy-(14β-O-(4-oxo-butyric acid-(3-phenylsulfonyl-1,2,5-oxadiazole-2-oxide-4) oxyethyl))-15-oxo-7,20-epoxy-16-kaurene* (**9a**). Yield 41%, m.p. 93–95 °C; ^1^H-NMR (CDCl_3_), *δ* (ppm) 1.12 (3H, s, –CH_3_), 1.25 (3H, s, –CH_3_), 3.16 (1H, d, *J* = 9.6 Hz, 13-CH), 3.49 (1H, m, 1-CH), 3.76 (1H, m, 6-CH), 4.02(1H, s, 1-OH), 4.06, 4.30 (each 1H, dd, *J*_A_ = *J*_B_ = 10.2 Hz, 20-CH_2_), 4.51 (2H, t, *J* = 4.5 Hz, –CH_2_), 4.62 (2H, t, *J* = 4.8 Hz, –CH_2_), 5.53 (1H, s, 17-CH_2_), 5.92 (1H, s, 14-CH), 6.04 (1H, d, *J* = 10.8 Hz, 6-OH), 6.15 (1H, s, 17-CH_2_), 7.64 (2H, t, *J* = 7.2 Hz, Ar-H), 7.77 (1H, t, *J* = 7.5 Hz, Ar-H), 8.07 (2H, d, *J* = 8.1 Hz, Ar-H); MS(ESI) *m/z*: 755.4 [M*+*Na]^+^; HR-MS (ESI, M+Na) *m/z*: calcd for C_34_H_40_N_2_NaO_14_S: 755.2092, found 755.2095.

*ent-1α,6β,7β-Trihydroxy-(14β-O-(4-oxobutyric acid-(3-phenylsulfonyl-1,2,5-oxadiazole-2-oxide-4)-oxypropyl))-15-oxo-7,20-epoxy-16-kaurene* (**9b**). Yield 34%, m.p. 86–88 °C; IR *υ*_max_ 3419, 2955, 2025, 1736, 1615, 1554, 1451, 1359, 733, 686 cm^−1^; ^1^H-NMR (CDCl_3_), *δ* (ppm) 1.11 (6H, s, –CH_3_), 3.14 (1H, d, *J* = 9.6 Hz, 13-CH), 3.49 (1H, m, 1-CH), 3.74 (1H, m, 6-CH), 4.07 (1H, s, 1-OH), 4.28 (2H, m, –CH_2_), 4.06, 4.30 (each 1H, dd, *J*_A_ = *J*_B_ = 10.2 Hz, 20-CH_2_), 4.50 (2H, t, *J* = 6.0 Hz, –CH_2_), 5.52 (1H, s, 17-CH_2_), 5.89 (1H, s, 14-CH), 6.07 (1H, d, *J* = 10.8 Hz, 6-OH), 6.15 (1H, s, 17-CH_2_), 7.63 (2H, t, *J* = 7.8 Hz, Ar-H), 7.77 (1H, t, *J* = 7.2 Hz, Ar-H), 8.06 (2H, d, *J* = 7.2 Hz, Ar-H); MS(ESI) *m/z*: 764.3 [M*+*NH_4_]^+^, 747.3 [M*+*H]^+^, 781.4 [M*+*Cl]^−^; HR-MS (ESI, M+Na) *m/z*: calcd for C_35_H_42_N_2_NaO_14_S: 769.2249, found 769.2254.

*ent-1α,6β,7β-Trihydroxy-(14β-O-(4-oxobutyric acid-(3-phenylsulfonyl-1,2,5-oxadiazole-2-oxide-4)-oxybutyl))-15-oxo-7,20-epoxy-16-kaurene* (**9c**). Yield 48%, m.p. 83–85 °C; IR *υ*_max_ 3417, 2955, 2025, 1734, 1635, 1554, 1451, 1367, 733, 686 cm^–1^; ^1^H-NMR (CDCl_3_), *δ* (ppm) 1.11 (6H, s, –CH_3_), 3.15 (1H, d, *J* = 9.6 Hz, 13-CH), 3.48 (1H, m, 1-CH), 3.75 (1H, m, 6-CH), 4.07 (1H, s, 1-OH), 4.17 (2H, m, –CH_2_), 4.05, 4.29 (each 1H, dd, *J*_A_ = *J*_B_ = 11.1 Hz, 20-CH_2_), 4.45 (2H, t, *J* = 6.0 Hz, –CH_2_), 5.52 (1H, s, 17-CH_2_), 5.90 (1H, s, 14-CH), 6.06 (1H, d, *J* = 10.8 Hz, 6–OH), 6.15 (1H, s, 17-CH_2_), 7.63 (2H, t, *J* = 7.5 Hz, Ar-H), 7.77 (1H, t, *J* = 7.2 Hz, Ar-H), 8.06 (2H, d, *J* = 7.2 Hz, Ar-H); MS(ESI) *m/z*: 778.2 [M*+*NH_4_]^+^, 761.1 [M*+*H]^+^, 795.2 [M*+*Cl]^−^; HR-MS (ESI, M+Na) *m/z*: calcd for C_36_H_44_N_2_NaO_14_S: 783.2405, found 783.2411.

*ent-1α,6β,7β-Trihydroxy-(14β-O-(5-oxo-pentanoic acid-(3-phenylsulfonyl-1,2,5-oxadiazole-2-oxide-4)-oxyethyl))-15-oxo-7,20-epoxy-16-kaurene* (**9d**). Yield 45%, m.p. 86–89 °C; IR *υ*_max_ 3405, 2952, 2025, 1739, 1618, 1554, 1451, 1360, 732, 676 cm^–1^; ^1^H-NMR (CDCl_3_), *δ* (ppm) 1.10 (6H, s, –CH_3_), 3.16 (1H, d, *J* = 9.9 Hz, 13-CH), 3.48 (1H, m, 1-CH), 3.73 (1H, m, 6-CH), 4.14 (1H, s, 1-OH), 4.05, 4.30 (each 1H, dd, *J*_A_ = *J*_B_ = 10.2 Hz, 20-CH_2_), 4.47 (2H, m, –CH_2_), 4.61 (2H, m, –CH_2_), 5.50 (1H, s, 17-CH_2_), 5.87 (1H, s, 14-CH), 6.06 (1H, d, *J* = 10.5 Hz, 6-OH), 6.13 (1H, s, 17-CH_2_), 7.63 (2H, t, *J* = 7.5 Hz, Ar-H), 7.77 (1H, t, *J* = 7.5 Hz, Ar-H), 8.06 (2H, d, *J* = 7.8 Hz, Ar-H); MS(ESI) *m/z*: 764.0 [M*+*NH_4_]^+^, 747.1 [M*+*H]^+^, 781.2 [M*+*Cl]^−^; HR-MS (ESI, M+Na) *m/z*: calcd for C_35_H_42_N_2_NaO_14_S: 769.2249, found 769.225.

*ent-1α,6β,7β-Trihydroxy-(14β-O-(5-oxopentanoic acid-(3-phenylsulfonyl-1,2,5-oxadiazole-2-oxide-4)-oxypropyl))-15-oxo-7,20-epoxy-16-kaurene* (**9e**). Yield 42%, m.p. 80–82 °C; IR *υ*_max_ 3439, 2954, 2025, 1734, 1615, 1554, 1452, 1383, 733, 686 cm^–1^; ^1^H-NMR (CDCl_3_), *δ* (ppm) 1.11 (6H, s, –CH_3_), 3.16 (1H, d, *J* = 9.6 Hz, 13-CH), 3.50 (1H, m, 1-CH), 3.75 (1H, m, 6-CH), 4.07, 4.30 (each 1H, dd, *J*_A_ = *J*_B_ = 10.8 Hz, 20-CH_2_), 4.25 (2H, t, *J* = 6.0 Hz, –CH_2_), 4.50 (2H, t, *J* = 6.0 Hz, –CH_2_), 5.51 (1H, s, 17-CH_2_), 5.87 (1H, s, 14-CH), 6.05 (1H, d, *J* = 10.5 Hz, 6–OH), 6.14 (1H, s, 17-CH_2_), 7.63 (2H, t, *J* = 7.5 Hz, Ar-H), 7.77 (1H, t, *J* = 7.5 Hz, Ar-H), 8.05 (2H, d, *J* = 7.5 Hz, Ar-H); MS(ESI) *m/z*: 778.3 [M*+*NH_4_]^+^, 761.3 [M*+*H]^+^, 795.4 [M*+*Cl]^−^; HR-MS (ESI, M+Na) *m/z*: calcd for C_36_H_44_N_2_NaO_14_S: 783.2405, found 783.2419.

*ent-1α,6β,7β-Trihydroxy-(14β-O-(5-oxo-pentanoic acid-(3-phenylsulfonyl-1,2,5-oxadiazole-2-oxide-4)-oxybutyl))-15-oxo-7,20-epoxy-16-kaurene* (**9f**). Yield 37%, m.p. 92–94 °C; IR *υ*_max_ 3440, 2955, 2025, 1733, 1615, 1554, 1451, 1367, 732, 686 cm^–1^; ^1^H-NMR (CDCl_3_), *δ* (ppm) 1.11 (6H, s, –CH_3_), 3.39 (1H, d, *J* = 9.9 Hz, 13-CH), 3.50 (1H, m, 1-CH), 3.76 (1H, m, 6-CH), 4.06 (1H, s, 1-OH), 4.08, 4.34 (each 1H, dd, *J*_A_ = *J*_B_ = 10.2 Hz, 20-CH_2_), 4.46 (1H, m, –CH_2_), 4.57 (1H, m, –CH_2_), 4.58 (2H, m, –CH_2_), 5.56 (1H, s, 17-CH_2_), 6.05 (1H, d, *J* = 10.5 Hz, 6-OH), 6.07 (1H, s, 14-CH), 6.17 (1H, s, 17-CH_2_), 7.52 (2H, m, Ar-H), 7.57 (3H, m, Ar-H), 7.75 (2H, m, Ar-H), 8.06 (2H, d, *J* = 7.5 Hz, Ar-H); MS(ESI) *m/z*: 792.3 [M*+*NH_4_]^+^, 775.5 [M*+*H]^+^, 809.6 [M*+*Cl]^−^; HR-MS (ESI, M+Na) *m/z*: calcd for C_37_H_46_N_2_NaO_14_S: 797.2562, found 797.2565.

*ent-1α,6β,7β-Trihydroxy-(14β-O-(2-formyl benzoic acid-(3-phenylsulfonyl-1,2,5-oxadiazole-2-oxide-4)-oxyethyl))-15-oxo-7,20-epoxy-16-kaurene* (**9g**). Yield 53%, m.p. 123–125 °C; IR *υ*_max_ 3392, 2951, 2025, 1714, 1618, 1553, 1451, 1363, 739, 685 cm^–1^; ^1^H-NMR (CDCl_3_), *δ* (ppm) 1.09 (6H, s, –CH_3_), 3.37 (1H, d, *J* = 9.6 Hz, 13-CH), 3.50 (1H, m, 1-CH), 3.72 (1H, m, 6-CH), 4.05 (1H, s, 1–OH), 4.07, 4.36 (each 1H, dd, *J*_A_ = *J*_B_ = 10.2 HZ, 20-CH_2_), 4.67 (2H, m, –CH_2_), 4.76 (2H, m, –CH_2_), 5.54 (1H, s, 17-CH_2_), 6.04 (1H, d, *J* = 12.0 Hz, 6-OH), 6.07 (1H, s, 14-CH), 6.14 (1H, s, 17-CH_2_), 7.46 (2H, t, *J* = 7.8 Hz, Ar-H), 7.58 (4H, m, Ar-H), 7.78 (1H, m, Ar-H), 8.01 (2H, d, *J* = 7.5 Hz, Ar-H); MS(ESI) *m/z*: 798.3 [M*+*NH_4_]^+^, 781.2 [M*+*H]^+^, 815.3 [M*+*Cl]^−^; HR-MS (ESI, M+Na) *m/z*: calcd for C_38_H_40_N_2_NaO_14_S: 803.2092, found 755.2093.

*ent-1α,6β,7β-Trihydroxy-(14β-O-(2-formyl benzoic acid-(3-phenylsulfonyl-1,2,5-oxadiazole-2-oxide-4)-oxypropyl))-15-oxo-7,20-epoxy-16-kaurene* (**9h**). Yield 47%, m.p. 113–115 °C; IR *υ*_max_ 3418, 2954, 2025, 1714, 1616, 1554, 1451, 1384, 736, 685 cm^–1^; ^1^H-NMR (CDCl_3_), *δ* (ppm) 1.11 (6H, s, –CH_3_), 3.39 (1H, d, *J* = 9.9 Hz, 13-CH), 3.50 (1H, m, 1-CH), 3.76 (1H, m, 6-CH), 4.06 (1H, s, 1-OH), 4.08, 4.34 (each 1H, dd, *J*_A_ = *J*_B_ = 10.2 Hz, 20-CH_2_), 4.46 (1H, m, –CH_2_), 4.57 (1H, m, –CH_2_), 4.58 (2H, m, –CH_2_), 5.56 (1H, s, 17-CH_2_), 6.05 (1H, d, *J* = 10.5 Hz, 6-OH), 6.07 (1H, s, 14-CH), 6.17 (1H, s, 17-CH_2_), 7.52 (2H, m, Ar-H), 7.57 (3H, m, Ar-H), 7.75 (2H, m, Ar-H), 8.06 (2H, d, *J* = 7.5 Hz, Ar-H); MS(ESI) **m/z**: 812.3 [M*+*NH_4_]^+^, 795.3 [M*+*H]^+^, 829.4 [M*+*Cl]^−^; HR-MS (ESI, M+Na) **m/z**: calcd for C_39_H_42_N_2_NaO_14_S: 817.2249, found 755.2252.

*ent-1α,6β,7β-Trihydroxy-(14β-O-(2-formylbenzoic acid-(3-phenylsulfonyl-1,2,5-oxadiazole-2-oxide-4)-oxybutyl))-15-oxo-7,20-epoxy-16-kaurene* (**9i**). Yield 50%, m.p. 108–110 °C; IR *υ*_max_ 3384, 2952, 2025, 1715, 1615, 1553, 1450, 1368, 734, 685 cm^–1^; ^1^H-NMR (CDCl_3_), *δ* (ppm) 1.10 (6H, s, –CH_3_), 3.32 (1H, d, *J* = 9.9 Hz, 13-CH), 3.50 (1H, m, 1-CH), 3.76 (1H, m, 6-CH), 4.08, 4.34 (each 1H, dd, *J*_A_ = *J*_B_ = 8.4 HZ, 20-CH_2_), 4.44 (2H, m, –CH_2_), 4.50 (2H, t, *J* = 5.4 Hz, –CH_2_), 5.30 (1H, s, 1-OH), 5.56 (1H, s, 17-CH_2_), 6.04 (1H, d, *J* = 10.5 Hz, 6-OH), 6.09 (1H, s, 14-CH), 6.61 (1H, s, 17-CH_2_), 7.53 (3H, m, Ar-H), 7.61 (2H, m, Ar-H), 7.76 (2H, m, Ar-H), 8.06 (2H, d, *J* = 7.8 Hz, Ar-H); MS(ESI) *m/z*: 826.1 [M*+*NH_4_]^+^, 809.0 [M*+*H]^+^, 843.3 [M*+*Cl]^−^; HR-MS (ESI, M+Na) *m/z*: calcd for C_40_H_44_N_2_NaO_14_S: 831.2405, found 831.2411.

*ent-(1α-O-Acetyl)-6β,7β-dihydroxy-(14β-O-(4-oxobutyric acid-(3-phenylsulfonyl-1,2,5-oxadiazole-2-oxide-4)-oxyethyl))-15-oxo-7,20-epoxy-16-kaurene* (**13a**). Yield 40%, m.p. 105–107 °C; IR *υ*_max_ 3384, 2958, 2025, 1739, 1618, 1554, 1452, 1363, 732, 686 cm^–1^; ^1^H-NMR (CDCl_3_), *δ* (ppm) 1.12 (6H, s, –CH_3_), 2.17 (3H, s, –CH_3_), 3.13 (1H, d, *J* = 9.6 Hz, 13-CH), 3.76 (1H, m, 6-CH), 4.17, 4.28 (each 1H, dd, *J*_A_ = *J*_B_ = 10, 5 Hz, 20-CH_2_), 4.51 (2H, m, –CH_2_), 4.61 (1H, m, 1-CH), 4.62 (2H, m, –CH_2_), 5.52 (1H, s, 17-CH_2_), 5.87 (1H, s, 14-CH), 6.09 (1H, d, *J* = 9.3 Hz, 6–OH), 6.15 (1H, s, 17-CH_2_), 7.63 (2H, t, *J* = 7.2 Hz, Ar-H), 7.91 (1H, t, *J* = 7.8 Hz, Ar-H), 8.07 (2H, d, *J* = 7.2 Hz, Ar-H); MS(ESI) *m/z*: 775.3 [M*+*H]^+^, 809.4 [M*+*Cl]^−^; HR-MS (ESI, M+Na) *m/z*: calcd for C_36_H_42_N_2_NaO_15_S: 797.2198, found 797.2207.

*ent-(1α-O-Acetyl)-6β,7β-dihydroxy-(14β-O-(4-oxobutyric acid-(3-phenylsulfonyl-1,2,5-oxadiazole-2-oxide-4)-oxypropyl))-15-oxo-7,20-epoxy-16-kaurene* (**13b**). Yield 51%, m.p. 95–97 °C; IR *υ*_max_ 3383, 2957, 2025, 1738, 1615, 1554, 1452, 1373, 733, 686 cm^–1^; ^1^H-NMR (CDCl_3_), *δ* (ppm) 1.12 (6H, s, –CH_3_), 1.99 (3H, s, –CH_3_), 3.12 (1H, d, *J* = 9.6 Hz, 13-CH), 3.77 (1H, m, 6-CH), 4.31 (2H, m, –CH_2_), 4.17, 4.34 (each 1H, dd, *J*_A_ = *J*_B_ = 10.2 Hz, 20-CH_2_), 4.51 (2H, t, *J* = 6.0 Hz, –CH_2_), 4.62 (1H, m, 1-CH), 5.52 (1H, s, 17-CH_2_), 5.85 (1H, s, 14-CH), 6.12 (1H, d, *J* = 11.1 Hz, 6–OH), 6.15 (1H, s, 17-CH_2_), 7.64 (2H, t, *J* = 7.8 Hz, Ar-H), 7.77 (1H, t, *J* = 7.5 Hz, Ar-H), 8.06 (2H, d, *J* = 7.2 Hz, Ar-H); MS(ESI) *m/z*: 806.3 [M*+*NH_4_]^+^, 789.3 [M*+*H]^+^, 823.3 [M*+*Cl]^−^; HR-MS (ESI, M+Na) *m/z*: calcd for C_37_H_44_N_2_NaO_15_S: 811.2355, found 811.2362.

*ent-(1α-O-Acetyl)-6β,7β-dihydroxy-(14β-O-(4-oxobutyric acid-(3-phenylsulfonyl-1,2,5-oxadiazole-2-oxide-4)-oxybutyl))-15-oxo-7,20-epoxy-16-kaurene* (**13c**). Yield 48%, m.p. 109–111 °C; IR *υ*_max_ 3385, 2957, 2025, 1737, 1616, 1554, 1451, 1371, 733, 686 cm^–1^; ^1^H-NMR (CDCl_3_), *δ* (ppm) 1.12 (6H, s, –CH_3_), 1.99 (3H, s, CH_3_), 3.13 (1H, d, *J* = 9.6 Hz, 13-CH), 3.78 (1H, m, 6-CH), 4.19 (2H, m, –CH_2_), 4.11, 4.27 (each 1H, dd, *J*_A_ = *J*_B_ = 10.5 Hz, 20-CH_2_), 4.46 (2H, t, *J* = 7.5 Hz, –CH_2_), 4.62 (1H, m, 1-CH), 5.53 (1H, s, 17-CH_2_), 5.88 (1H, s, 14-CH), 6.11 (1H, d, *J* = 10.5 Hz, 6–OH), 6.16 (1H, s, 17-CH_2_), 7.64 (2H, t, *J* = 7.5 Hz, Ar-H), 7.78 (1H, t, *J* = 7.2 Hz, Ar-H), 8.07 (2H, d, *J* = 7.8 Hz, Ar-H); MS(ESI) *m/z*: 820.4 [M*+*NH_4_]^+^, 803.3 [M*+*H]^+^, 837.3 [M*+*Cl]^−^; HR-MS (ESI, M+Na) *m/z*: calcd for C_38_H_46_N_2_NaO_15_S: 825.2511, found 825.2525.

*ent-(1α-O-Acetyl)-6β,7β-dihydroxy-(14β-O-(4-oxopentanoic acid-(3-phenylsulfonyl-1,2,5-oxadiazole-2-oxide-4)-oxyethyl))-15-oxo-7,20-epoxy-16-kaurene* (**13d**). Yield 42%, m.p. 92–94 °C; IR *υ*_max_ 3530, 3386, 2956, 2025, 1738, 1618, 1553, 1451, 731, 686 cm^–1^; ^1^H-NMR (CDCl_3_), *δ* (ppm) 1.12 (6H, s, CH_3_), 1.99 (3H, s, –CH_3_), 3.16 (1H, d, *J* = 9.9 Hz, 13-CH), 3.75 (1H, m, 6-CH), 4.17, 4.27 (each 1H, dd, *J*_A_ = *J*_B_ = 10.5 Hz, 20-CH_2_), 4.49 (2H, m, –CH_2_), 4.61 (2H, m, –CH_2_), 5.50 (1H, s, 17-CH_2_), 5.83 (1H, s, 14-CH), 6.06 (1H, d, *J* = 9.6 Hz, 6–OH), 6.13 (1H, s, 17-CH_2_), 7.63 (2H, t, *J* = 7.8 Hz, Ar-H), 7.76 (1H, t, *J* = 7.5 Hz, Ar-H), 8.06 (2H, d, *J* = 7.5 Hz, Ar-H); MS(ESI) *m/z*: 806.4 [M*+*NH_4_]^+^, 789.2 [M*+*H]^+^, 823.3 [M*+*Cl]^−^; HR-MS (ESI, M+Na) *m/z*: calcd for C_37_H_44_N_2_NaO_15_S: 811.2355, found 811.2367.

*ent-(1α-O-Acetyl)-6β,7β-dihydroxy-(14β-O-(5-oxopentanoic acid-(3-phenylsulfonyl-1,2,5-oxadiazole-2-oxide-4)-oxypropyl))-15-oxo-7,20-epoxy-16-kaurene* (**13e**). Yield 36%, m.p. 86–88 °C; IR *υ*_max_ 3421, 2958, 2025, 1737, 1616, 1554, 1452, 1374, 732, 686 cm^–1^; ^1^H-NMR (CDCl_3_), *δ* (ppm) 1.12 (6H, s, –CH_3_), 2.19 (3H, s, –CH_3_), 3.16 (1H, d, *J* = 10.2 Hz, 13-CH), 3.77 (1H, m, 6-CH), 4.20, 4.38 (each 1H, dd, *J*_A_ = *J*_B_ = 10.5 Hz, 20-CH_2_), 4.26 (2H, t, *J* = 6.0 Hz, –CH_2_), 4.50 (2H, t, *J* = 6.0 Hz, –CH_2_), 4.62 (1H, m, 1-CH), 5.52 (1H, s, 14-CH), 5.83 (1H, s, 17-CH_2_), 6.06 (1H, d, *J* = 10.5 Hz, 6–OH), 6.15 (1H, s, 17-CH_2_), 7.63 (2H, t, *J* = 7.5 Hz, Ar-H), 7.77 (1H, t, *J* = 7.2 Hz, Ar-H), 8.06 (2H, d, *J* = 7.5 Hz, Ar-H); MS(ESI) *m/z*: 803.3 [M*+*H]^+^, 837.4 [M*+*Cl]^−^; HR-MS (ESI, M+Na) *m/z*: calcd for C_38_H_46_N_2_NaO_15_S: 825.2511, found 825.2523.

*ent-(1α-O-Acetyl)-6β,7β-dihydroxy-(14β-O-(5-oxopentanoic acid-(3-phenylsulfonyl-1,2,5-oxadiazole-2-oxide-4)-oxybutyl))-15-oxo-7,20-epoxy-16-kaurene* (**13f**). Yield 40%, m.p. 98–101 °C; IR *υ*_max_ 3394, 2957, 2025, 1737, 1617, 1554, 1451, 1373, 732, 686 cm^–1^; ^1^H-NMR (CDCl_3_), *δ* (ppm) 1.12 (6H, s, –CH_3_), 2.35 (3H, s, –CH_3_), 3.17 (1H, d, *J* = 9.3 Hz, 13-CH), 3.76 (1H, m, 6-CH), 4.20 (2H, t, *J* = 6.0 Hz, –CH_2_), 4.18, 4.27 (each 1H, dd, *J*_A_ = *J*_B_ = 10.2 Hz, 20-CH_2_), 4.46 (2H, t, *J* = 5.7 Hz, –CH_2_), 4.61 (1H, m, 1-CH), 5.52 (1H, s, 17-CH_2_), 5.83 (1H, s, 14-CH), 6.07 (1H, d, *J* = 10.2 Hz, 6–OH), 6.15 (1H, s, 17-CH_2_), 7.63 (2H, t, *J* = 7.2 Hz, Ar-H), 7.74 (1H, t, *J* = 7.8 Hz, Ar-H), 8.05 (2H, d, *J* = 7.2 Hz, Ar-H); MS(ESI) *m/z*: 834.4 [M*+*NH_4_]^+^, 817.3 [M*+*H]^+^, 851.3 [M*+*Cl]^−^; HR-MS (ESI, M+Na) *m/z*: calcd for C_39_H_48_N_2_NaO_15_S: 839.2668, found 839.2679.

*ent-(1α-O-Acetyl)-6β,7β-dihydroxy-(14β-O-(3-formylbenzoic acid-(3-phenylsulfonyl-1,2,5-oxadiazole-2-oxide-4)-oxyethyl))-15-oxo-7,20-epoxy-16-kaurene* (**13g**). Yield 46%, m.p. 154–156 °C; IR *υ*_max_ 3383, 2957, 2025, 1736, 1617, 1554, 1451, 1364, 739, 685 cm^–1^; ^1^H-NMR (CDCl_3_), *δ* (ppm) 1.12 (6H, s, –CH_3_), 2.01 (3H, s, CH_3_), 3.36 (1H, d, *J* = 9.6 Hz, 13-CH), 3.75 (1H, m, 6-CH), 4.19, 4.36 (each 1H, dd, *J*_A_ = *J*_B_ = 10.5 Hz, 20-CH_2_), 4.64 (2H, m, –CH_2_), 4.79 (2H, m, –CH_2_), 4.82 (1H, m, 1-CH), 5.54 (1H, s, 17-CH_2_), 6.02 (1H, s, 14-CH), 6.03 (1H, d, *J* = 10.5 Hz, 6–OH), 6.15 (1H, s, 17-CH_2_), 7.44 (2H, t, *J* = 7.5 Hz, Ar-H), 7.79 (1H, m, Ar-H), 7.58 (4H, m, Ar-H), 8.03 (2H, d, *J* = 7.8 Hz, Ar-H); MS(ESI) *m/z*: 840.2 [M*+*NH_4_]^+^, 823.2 [M*+*H]^+^, 857.3 [M*+*Cl]^−^; HR-MS (ESI, M+Na) *m/z*: calcd for C_40_H_42_N_2_NaO_15_S: 845.2198, found 845.2208.

*ent-(1α-O-Acetyl)-6β,7β-dihydroxy-(14β-O-(3-formylbenzoic acid-(3-phenylsulfonyl-1,2,5-oxadiazole-2-oxide-4)-oxypropyl))-15-oxo-7,20-epoxy-16-kaurene* (**13h**). Yield 52%, m.p. 136–138 °C; IR *υ*_max_ 3379, 2957, 2025, 1735, 1616, 1554, 1451, 1374, 738, 685 cm^–1^; ^1^H-NMR (CDCl_3_), *δ* (ppm) 1.12 (6H, s, –CH_3_), 2.02 (3H, s, –CH_3_), 3.33 (1H, d, *J* = 9.6 Hz, 13-CH), 3.78 (1H, m, 6-CH), 4.22, 4.34 (each 1H, dd, *J*_A_ = *J*_B_ = 8.7 Hz, 20-CH_2_), 4.45 (1H, m, –CH_2_), 4.57 (1H, m, –CH_2_), 4.61 (2H, m, –CH_2_), 4.64 (1H, m, 1-CH), 5.56 (1H, s, 17-CH_2_), 6.02 (1H, s, 14-CH), 6.10 (1H, d, *J* = 10.5 Hz, 6–OH), 6.17 (1H, s, 17-CH_2_), 7.53 (2H, m, Ar-H), 7.62 (3H, m, Ar-H), 7.72 (2H, m, Ar-H), 8.09 (2H, d, *J* = 7.8 Hz, Ar-H); MS(ESI) *m/z*: 854.3 [M*+*NH_4_]^+^, 837.2 [M*+*H]^+^, 871.3 [M*+*Cl]^−^; HR-MS (ESI, M+Na) *m/z*: calcd for C_41_H_44_N_2_NaO_15_S: 859.2355, found 859.2368.

*ent-(1α-O-Acetyl)-6β,7β-dihydroxy-(14β-O-(2-formylbenzoic acid-(3-phenylsulfonyl-1,2,5-oxadiazole-2-oxide-4)-oxybutyl))-15-oxo-7,20-epoxy-16-kaurene* (**13i**). Yield 45%, m.p. 116–118 °C; IR *υ*_max_ 3382, 2958, 2025, 1726, 1616, 1553, 1451, 1373, 733, 685 cm^–1^; ^1^H-NMR (CDCl_3_), *δ* (ppm) 1.12 (6H, s, –CH_3_), 1.97 (3H, s, –CH_3_), 3.45 (1H, d, *J* = 9.9 Hz, 13-CH), 3.77 (1H, m, 6-CH), 4.21, 4.33 (each 1H, dd, *J*_A_ = *J*_B_ = 10.5 Hz, 20-CH_2_), 4.43 (2H, m, –CH_2_), 4.50 (2H, t, *J* = 5.7 Hz, –CH_2_), 4.64 (1H, m, 1-CH), 5.57 (1H, s, 17-CH_2_), 6.07 (1H, s, 14-CH), 6.07 (1H, d, *J* = 10.2 Hz, 6–OH), 6.16 (1H, s, 17-CH_2_), 7.54 (3H, m, Ar-H), 7.61 (2H, t, *J* = 7.8 Hz, Ar-H), 7.77 (2H, m, Ar-H), 8.06 (2H, d, J = 7.2 Hz, Ar-H); MS(ESI) *m/z*: 868.3 [M*+*NH_4_]^+^, 885.4 [M*+*Cl]^−^; HR-MS (ESI, M+Na) *m/z*: calcd for C_42_H_46_N_2_NaO_15_S: 873.2511, found 873.2527. 

### 3.2. *In Vitro* MTT Assay

The MTT assay was employed as an *in vitro* cytotoxicity assay, which was performed in 96-well plates. Test cells at the log phase of their growth cycle (5 × 10^4^ cell/mL) were added to each well (100 µL/well), then treated in three replicates at various concentrations of the samples (0.39–100 µg/mL), and incubated for 24 h at 37 °C in a humidified atmosphere of 5% CO_2_. After 72 h, 20 µL of MTT solution (5 mg/mL) per well was added to each cultured medium, which was incubated for further 4 h. Then, DMSO was added to each well (150 µL/well). After 10 min at room temperature, the OD of each well was measured on a Microplate Reader (BIO-RAD instruments Inc. No. 550, Hercules, CA, USA) at a wavelength of 490 nm. In these experiments, the negative reference was 0.1% DMSO, and taxol was used as the positive reference with the concentration of 10 µg/mL.

### 3.3. NO-Releasing Test: Nitrate/Nitrite Measurement *in Vitro*

The levels of nitrate/nitrite formed from individual compounds were determined by the colorimetric assay using the nitrate/nitrite colorimetric assay kit, in triplicate with 100 *μ*M of individual compounds for 0–60 min according to the manufacturer's instructions (Beyotime, Shanghai, China). The lysates were mixed with Griess for 40 min and centrifugalized for 10 min, and then followed by measuring at 540 nm, similar as previously reported [13,14,15,16,17].

## 4. Conclusions

In summary, a series of novel furoxan/oridonin hybrids were synthesized and tested for anti-proliferative activity against four human cancer cell lines by an *in vitro* MTT assay. Among them, compound **9h** exhibited the most potential anti-tumor activity against all test cell lines. The preliminary SAR of the target compounds was discussed based on the experimental data obtained. Furthermore, more than 15 μmol/L NO were produced by all target compounds at the 60 min time point, and the results showed that higher levels of NO releasing produced stronger anti-proliferative activity, so high levels of NO release by these hybrids could play a role in growth inhibition activity. These results suggested that NO-donor/natural product hybrids may provide a promising approach for the discovery of novel anti-tumor agents. Further studies on the structure modification of these hybrids and the mechanism of the derivatives are currently in progress and will be reported in due course.
